# Mol­ecular and crystal structure of (*Z*)-*N*-allyl-3-(allyl­imino)-3*H*-isoindol-1-amine

**DOI:** 10.1107/S2056989026009448

**Published:** 2026-09-30

**Authors:** Volodymyr A. Tkachuk, Svitlana V. Shishkina, Olga V. Hordiyenko

**Affiliations:** a National Taras Shevchenko University of Kyiv Faculty of Chemistry 64/13 Volodymyrs'ka str 01601 Kyiv Ukraine; b Institute of Organic Chemistry, NAS of Ukraine, Akademik Kukhar Street 5, Kyiv 02094, Ukraine; Vienna University of Technology, Austria

**Keywords:** crystal structure, 3-imino-3*H*-isoindol-1-amine derivatives, tautomerism, disorder, hydrogen-bonding

## Abstract

The 3-imino-3*H*-isoindol-1-amine tautomeric form of (*Z*)-*N*-allyl-3-(allyl­imino)-3*H*-isoindol-1-amine, C_14_H_15_N_3_, was confirmed by a single-crystal X-ray diffraction study. The distribution of electron density within the —NH—C=N—C=N— conjugated fragment corresponds to that found in previously studied *N*,*N*′-disubstituted 3-imino-3*H*-isoindol-1-amines. The two mol­ecules in the asymmetric unit of the title compound differ in the conformation of the allyl substituents. In one of these mol­ecules, the two allyl fragments are disordered over two sets of sites in 0.628 (7):0.372 (7) and 0.755 (5):0.245 (5) ratios. In the crystal, the two unique mol­ecules are arranged alternately in infinite chains parallel to [001] due to the formation of inter­molecular N—H⋯N hydrogen bonds.

## Abstract 2

The title mol­ecule is present in its 3-imino-3*H*-isoindol-1-amine tautomeric form. In the crystal, inter­molecular N—H⋯N hydrogen bonds lead to the formation of infinite chains propagating parallel to [001].

## 1. Chemical context

**Figure 1:**
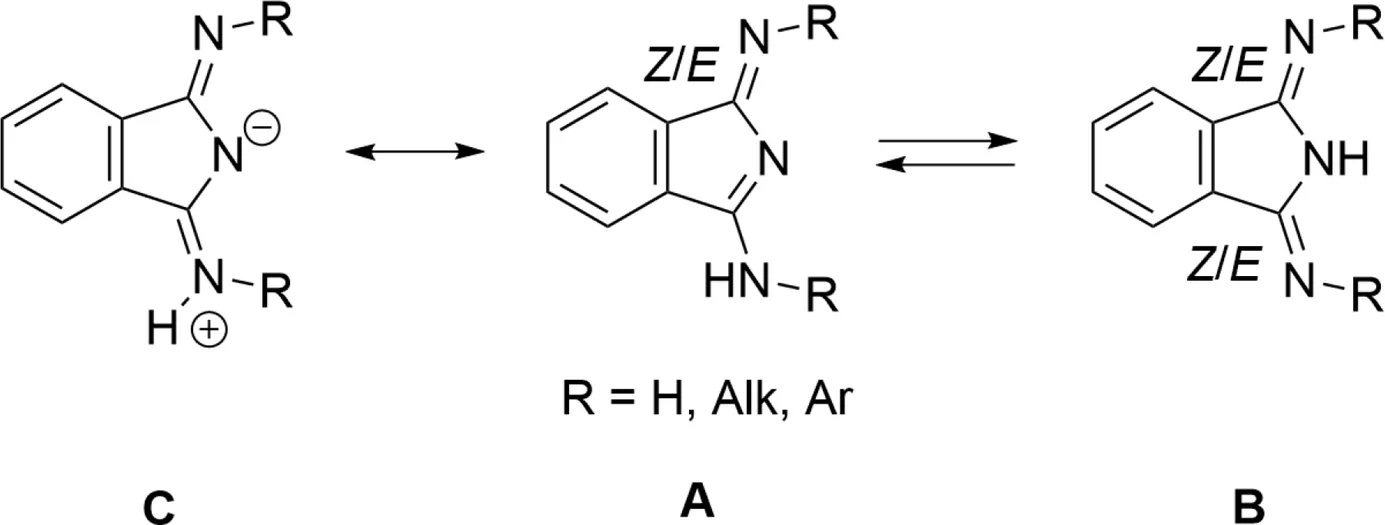
Zwitterionic and tautomeric forms of compound **1**.

Since the first synthesis and characterisation of 3-imino-3*H*-iso­indole-1-amine and its *N*,*N*′-disubstituted derivatives (Elvidge & Linstead, 1952 ▸; Clark *et al*., 1953 ▸), these compounds have been of inter­est as important inter­mediates in the synthesis of phthalocyanines and related compounds. They also exhibit structural features attributable to the presence of the conjugation fragment –NH—C=N–C=N–, in which tautomeric migration of the hydrogen atom between the exo- and endocyclic nitro­gen atoms can occur. This is evident from the presence of two tautomeric forms, *viz.* 3-imino-3*H*-isoindol-1-amine **A** and isoindoline-1,3-di­imine **B** (Fig. 1 ▸), which, depending on whether they are in the solid state or in solution, the medium and the substituents on the exocyclic nitro­gen atoms, can occur both as the starting compound and as *N*,*N*′-disubstituted derivatives (Negrebetskii *et al.*, 1983 ▸; Spiessens & Anteunis, 1984*a* ▸; Zhang *et al*., 2004*a* ▸).

Additionally, the presence of an exocyclic C=N double bond leads to the occurrence of *Z*/*E* isomerism (Negrebetskii *et al*., 1983 ▸; Spiessens & Anteunis, 1984*b* ▸; Zhang *et al*., 2004*b* ▸). In the solid state and in solution, both in the parent compound and in the *N*,*N*′-substituted derivatives, a significant polarization of the C=N bonds is realized, resulting in a significant contribution of the resonance structure **C** (Fig. 1 ▸; Shishkin *et al*., 2013 ▸).

A series of *N*,*N*′-dialkyl-substituted derivatives were synthesized using Siegl’s method from phthalo­nitrile and primary amines (Siegl, 1977 ▸; Tamgho *et al.*, 2013 ▸). As part of our investigations into *N*,*N*′-disubstituted derivatives of 3-imino-3*H*-isoindol-1-amine, we synthesized the title compound, (*Z*)-*N*-allyl-3-(allyl­imino)-3*H*-isoindol-1-amine, C_14_H_15_N_3_ (**1**), by the reaction of 1,1,3-tri­chloro-1*H*-iso­indole with N-allyl­amine, and report here its mol­ecular and crystal structure.
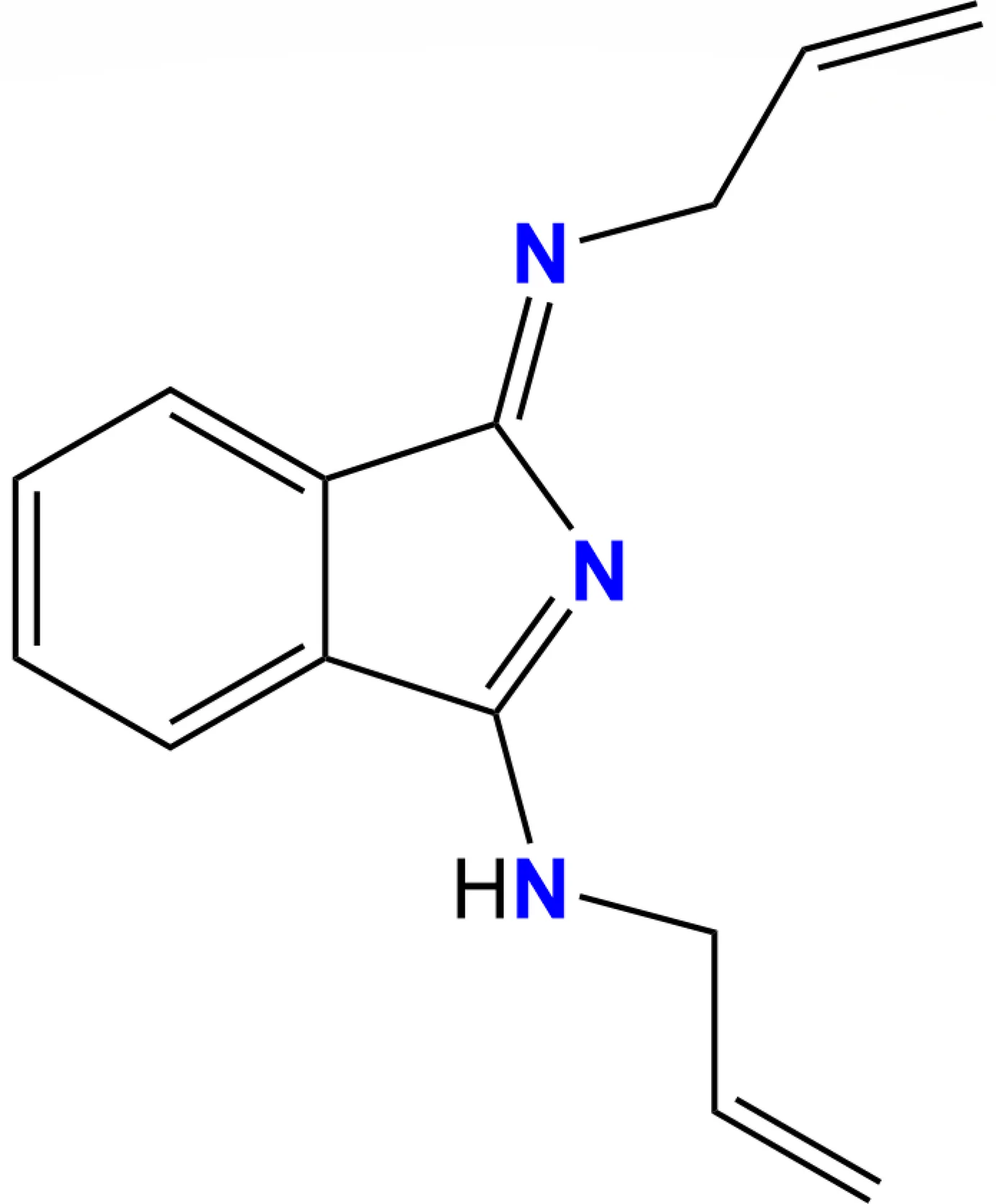


## 2. Structural commentary

**Figure 2:**
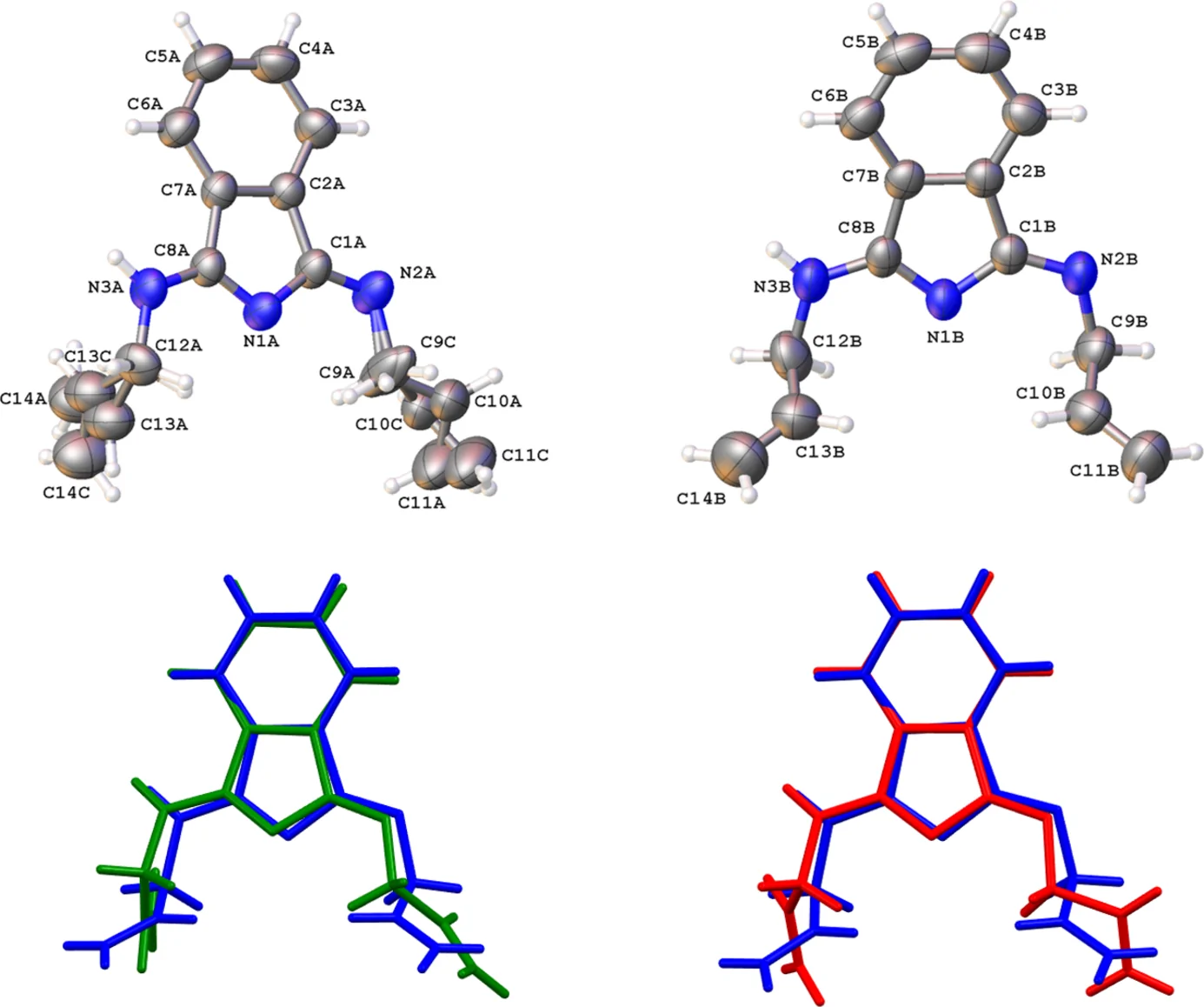
Mol­ecular structure of compound **1** with mol­ecules **a** and **b** present in the asymmetric unit (top row); displacement ellipsoids are drawn at the 50% probability level. Overlay of mol­ecules **1a**_major_component/**1b** (left) and **1a**_minor_component/**1b** (right); mol­ecule **1b** is shown in blue, **1a**_major_component in green and **1a**_minor_component in red (bottom row).

Two mol­ecules (**1a** and **1b**) are present in the asymmetric unit (Fig. 2 ▸). Analysis of bond lengths in the –N3H—C8=N1—C1=N2– fragment (Table 1 ▸) revealed that both mol­ecules exist in the tautomeric form **A** (Fig. 1 ▸) in the crystal. The C1—N2 and N1—C8 bond lengths are close to the mean value of 1.313 Å for C*sp*^2^=N(2) bonds (Bürgi & Dunitz, 1994 ▸; the number in brackets indicates the number of bonds formed by the nitro­gen atom), while the C1—N1 and C8—N3 bonds are comparable to the mean values of C*sp*^2^–N(2) and C*sp*^2^–N(3) bonds (1.376 Å and 1.339 Å; Bürgi & Dunitz, 1994 ▸), respectively. Mol­ecules **1a** and **1b** differ in the conformation of their allyl substituents. In mol­ecule **1b**, both allyl groups are arranged nearly orthogonal to the bicyclic fragment (the C1*B*—N2*B*—C9*B*—C10*B* and C8*B*—N3*B*—C12*B*—C13*B* torsion angles are −78.5 (3) and 82.1 (3)°). The terminal –C*sp*^2^=CH_2_ groups are rotated around the C9—C10 and C12—C13 single bonds [the N2*B*—C9*B*—C10*B*—C11*B* and N3*B*—C12*B*—C13*B*—C14*B* torsion angles are −116.6 (3) and 117.5 (4)°]. In mol­ecule **1a**, both allyl groups are disordered over two sets of sites due to rotation around the N2*A*—C9*A* or N3*A*—C12*A* bonds. The ratio of the conformers ***a*** and ***c*** for the C9–C10–C11 allyl group is 0.372 (7):0.628 (7), while the ratio of the conformers ***a*** and ***c*** for the C12–C13–C14 allyl group is 0.755 (5):0.245 (5). In conformer **1a*****_a***, the –C*sp*^2^=CH_2_ group is found in the *ap*-conformation relative to the C1*A*—N2*A* endocyclic bond and the C10=C11 double bond is turned in relation to the N2*A*—C9*A* bond [the C1*A*—N*2A*—C9*A*—C10*A* and N2*A*—C9*A*—C10*A*—C11*A* torsion angles are 175.7 (7) and −137 (1)°]. In conformer **1a*****_c***, the –C*sp*^2^=CH_2_ fragment is found in the *ac*-conformation relative to the C1*A*—N2*A* endocyclic bond, and its double bond is rotated around the C9C—C10C bond [the C1*A*—N2*A*—C9*C*—C10*C* and N2*A*—C9*C*—C10*C*—C11*C* torsion angles are 137.1 (5) and 149.6 (7)°]. The –C13=C14 fragment is arranged orthogonal in the two conformers [**1a*****_a*** and **1a*****_c***, the C8*A*—N3*A*—C12*A*—C13*A* and C8*A*— N3*A*—C12*A*—C13*C* torsion angles are −81.6 (4) and −96.8 (7)°] and the double bond is rotated around the C12*A*—C13*A*/C13*C* bond [the N3*A*—C12*A*—C13*A*—C14*A* and N3*A*—C12*A*—C13*C*—C14*C* torsion angles are −14.4 (6) and 142 (2)°].

## 3. Supra­molecular features

**Figure 3:**
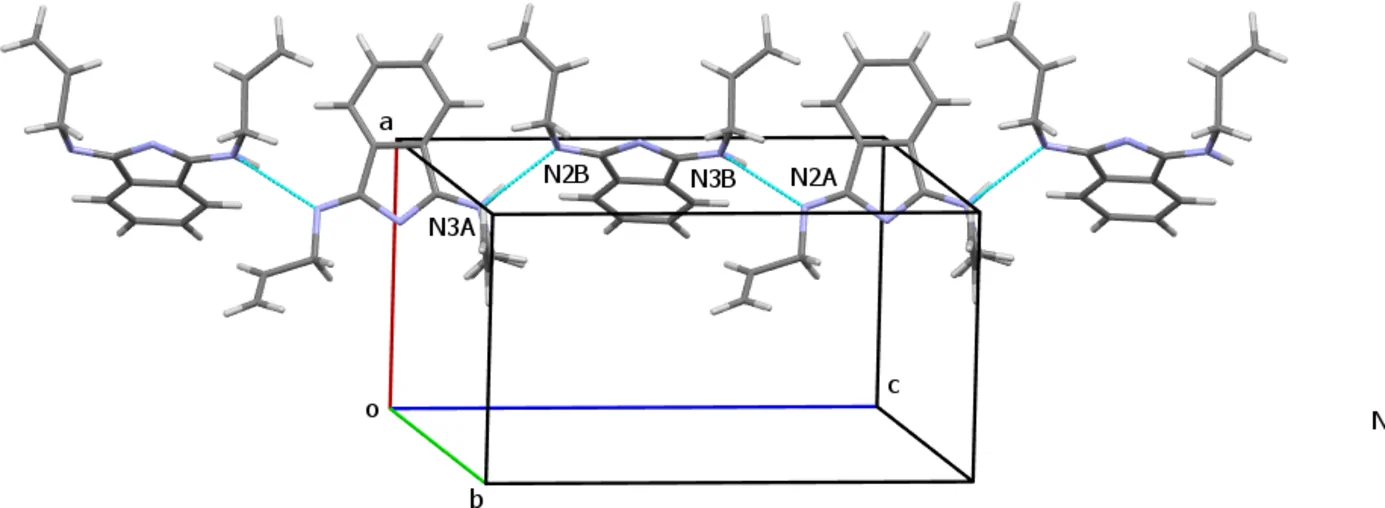
Chains of mol­ecules **1a** and **1b** extending parallel to [001]. Inter­molecular hydrogen bonds N—H⋯N are shown as blue dashed lines.

The title compound contains an NH group, which acts as a hydrogen donor, and two nitro­gen atoms, N1 and N2, which are capable of acting as hydrogen acceptors. However, one of these potential hydrogen acceptors, the N1 atom, is not available for hydrogen bonding due to the presence of bulky substituents at the neighboring atoms. As a result, only N3—H⋯N2 hydrogen bonds are found in the crystal (Table 2 ▸). These bonds form infinite chains parallel to [001], in which mol­ecules **1a** and **1b** alternate (Fig. 3 ▸). In addition to the classical N—H⋯N hydrogen bonds, only one weak C10—H⋯π(C6) inter­molecular inter­action is found in the crystal phase (Table 2 ▸). Despite the presence of an electron-rich bicyclic moiety in the mol­ecule, no stacking inter­actions are observed in the crystal.

## 4. Database survey

A search of the Cambridge Structure Database (CSD, version 6.00, last update April 2025; Groom *et al.*, 2016 ▸) revealed 40 structures containing the 3-imino-3*H*-isoindol-1-amine fragment. The *N*,*N*′-**un**substituted derivatives of 3-imino-3*H*-isoindol-1-amine (structures EGAKIK, EGAKOQ (Al-Shewiki *et al.*, 2024 ▸), EXUQAP, EXUQET (Zhang *et al.*, 2004**a* ▸)*, JARFAM (Lo & Ng, 2016 ▸), TIPVEV (Engle *et al.*, 2013 ▸)) proved to be the closest to the compound under study. The distribution of electron density within the –NH–C=N—C=N– fragment is identical in all these structures.

## 5. Synthesis and crystallization

Compound **1** was synthesized according to a literature procedure (Hordiyenko *et al.*, 2009 ▸, 2022 ▸). 1,1,3-Tri­chloro-1*H*-iso­indole (1.1 g, 5 mmol) was dissolved in anhydrous dioxane (5 ml), then *N*-allyl­amine (1.42 g, 25 mmol) was added dropwise upon stirring and cooling. A red oil formed, then the dioxane was evaporated under reduced pressure and the residual oil was washed with water (2 × 10 ml). After standing for one week in air, the oil crystallized in form of red crystals; yield 1.06 g (95%). Colourless single crystals were obtained by slow evaporation of a methano­lic solution of compound **1**.

^1^H NMR spectrum (400 MHz, CDCl_3_), *d*, ppm (*J*, Hz), **A** form (93%): 4.42 (*d*, *J* = 5.2 Hz, 4H, CH_2_), 5.14 (*d*, *J* = 10.0 Hz, 2H, *cis*-CH=), 5.27 (*d*, *J* = 17.2 Hz, 2H, *trans*-CH=), 6.06 (*m*, 2H, CH_2_—CH=), 6.5 (*br s*, 1H, NH), 7.38 (*m*, 2H, CH_Ar_), 7.38 (*m*, 2H, CH_Ar_). ^1^H NMR spectrum (400 MHz, CDCl_3_), *d*, ppm (*J*, Hz), **B** form (7%): 4.33 (*d*, *J* = 5.2 Hz, 4H, CH_2_), 7.33 (*d*, *J* = 7.6 Hz, 1H, CH_Ar_), 7.45–7.61 (*m*, 2H, CH_Ar_), 7.83 (*d*, *J* = 8.4 Hz, 1H, CH_Ar_).

^1^H NMR spectrum (400 MHz, DMSO-*d*_6_), *d*, ppm (*J*, Hz), **A** form (90%): 4.30 (*br s*, 4H, CH_2_), 5.86 (*d*, *J* = 10.0 Hz, 2H, *cis*-CH=), 5.23 (*d*, *J* = 16.8 Hz, 2H, *trans*-CH=), 5.99–6.06 (*m*, 2H, CH_2_-CH=), 7.48–7.50 (*m*, 2H, CH_Ar_), 7.74–7.82 (*m*, 2H, CH_Ar_). ^1^H NMR spectrum (400 MHz, CDCl_3_), *d*, ppm (*J*, Hz), **B** form (10%): 4.22 (*br s*, 4H, CH_2_), 5.86 (*m*, 2H, CH_2_—CH=), 7.69–7.71 (*m*, 2H, CH_Ar_), 7.89 (m, 2H, CH_Ar_).

^13^C NMR spectrum (100 MHz, CDCl_3_), *d*, ppm: 49.5 (CH_2_), 116.7, 117.6, 120.7, 121.9, 125.7, 129.4, 130.3, 135.0, 137.4, 167.1.

LC-MS: RT (min), *m*/*z* (%) = 0.951, 226.2 [68%, (*M* + H)^+^]; 1.019, 226.2 [21%, (*M* + H)^+^]. The presence of two independent peaks at *m*/*z* 226 but different retention times may be considered as a presence of two tautomeric forms **A**/**B** in a 3:1 ratio in solution during LS-MS analysis (RP C18, gradient elution of solvent H_2_O/CH_3_CN:1/99 to H_2_O/CH_3_CN:0/100, 0.1% formic acid). The ^1^H NMR spectra of compound **1** in CDCl_3_ and DMSO-*d*_6_ revealed two sets of resonances at room temperature corresponding to the **A** and **B** tautomers in a 90:10 ratio.

## 6. Refinement

Crystal data, data collection and structure refinement details are summarized in Table 3 ▸. Hydrogen atoms were placed at calculated positions and refined as riding with *U*_iso_(H) = 1.2*U*_eq_(C/N). The site occupation factors of the disordered allyl groups of mol­ecule A were refined as 0.628 (7):0.372 (7) and 0.755 (5):0.245 (5). The restrictions on the bonds lengths in the disordered fragments were applied as C*sp*^2^—C*sp*^3^ = 1.510 (2) Å, N(2)—C*sp*^3^ = 1.470 (2) Å, C*sp*^2^=C*sp*^2^ = 1.340 (2) Å.

## Supplementary Material

Crystal structure: contains datablock(s) I. DOI: 10.1107/S2056989026009448/wm5805sup1.cif

Structure factors: contains datablock(s) I. DOI: 10.1107/S2056989026009448/wm5805Isup3.hkl

CCDC reference: 2586660

Additional supporting information:  crystallographic information; 3D view; checkCIF report

## Figures and Tables

**Table 1:** Selected bond lengths (Å) in compound (**I**)

Bond	Mol­ecule **1a**	Mol­ecule **1b**
C1—N2	1.281 (3)	1.275 (3)
C1—N1	1.395 (3)	1.403 (4)
N1—C8	1.317 (3)	1.319 (3)
C8—N3	1.325 (3)	1.322 (3)

**Table 2:** Hydrogen-bond geometry (Å, °)

*D*—H⋯*A*	*D*—H	H⋯*A*	*D*⋯*A*	*D*—H⋯*A*
C10*A*—H10*A*⋯C6*B*^i^	0.93	2.74	3.571 (12)	149
N3*A*—H3*AA*⋯N2*B*^ii^	0.86	2.11	2.895 (3)	151
N3*B*—H3*BA*⋯N2*A*^i^	0.86	2.15	2.951 (3)	154

Symmetry codes: (i) 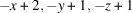; (ii) 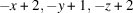.

**Table 3:** Experimental details

Crystal data	
Chemical formula	C_14_H_15_N_3_
*M*_r_	225.29
Crystal system, space group	Triclinic, *P* 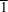
Temperature (K)	293
*a*, *b*, *c* (Å)	9.2429 (6), 11.1785 (8), 13.8791 (11)
α, β, γ (°)	73.461 (7), 87.231 (6), 67.950 (6)
*V* (Å^3^)	1271.36 (17)
*Z*	4
Radiation type	Mo *K*α
μ (mm^−1^)	0.07
Crystal size (mm)	0.27 × 0.23 × 0.12

Data collection	
Diffractometer	Xcalibur, Sapphire3
Absorption correction	Multi-scan (*CrysAlis PRO*; Rigaku OD, 2024 ▸)
*T*_min_, *T*_max_	0.806, 1.000
No. of measured, independent and observed [*I* > 2σ(*I*)] reflections	9706, 4463, 2711
*R*_int_	0.024
(sin θ/λ)_max_ (Å^−1^)	0.595

Refinement	
*R*[*F*^2^ > 2σ(*F*^2^)], *wR*(*F*^2^), *S*	0.059, 0.190, 1.05
No. of reflections	4463
No. of parameters	324
No. of restraints	118
H-atom treatment	H-atom parameters constrained
Δρ_max_, Δρ_min_ (e Å^−3^)	0.32, −0.25

Computer programs: *CrysAlis PRO* (Rigaku OD, 2024 ▸), *SHELXT* (Sheldrick, 2015*a* ▸), *SHELXL* (Sheldrick, 2015*b* ▸), *Mercury* (Macrae *et al.*, 2020 ▸) and *publCIF* (Westrip, 2010 ▸).

## supplementary crystallographic information

### (Z)-N-(Prop-2-en-1-yl)-3-(prop-2-en-1-ylimino)isoindol-1-amine Crystal data

**Table:**

C_14_H_15_N_3_	*Z* = 4
*M**_r_* = 225.29	*F*(000) = 480
Triclinic, *P*1	*D*_x_ = 1.177 Mg m^−^^3^
*a* = 9.2429 (6) Å	Mo *K*α radiation, λ = 0.71073 Å
*b* = 11.1785 (8) Å	Cell parameters from 3958 reflections
*c* = 13.8791 (11) Å	θ = 3.1–32.5°
α = 73.461 (7)°	µ = 0.07 mm^−^^1^
β = 87.231 (6)°	*T* = 293 K
γ = 67.950 (6)°	Bar, colourless
*V* = 1271.36 (17) Å^3^	0.27 × 0.23 × 0.12 mm

### (Z)-N-(Prop-2-en-1-yl)-3-(prop-2-en-1-ylimino)isoindol-1-amine Data collection

**Table:**

Xcalibur, Sapphire3 diffractometer	4463 independent reflections
Radiation source: Enhance (Mo) X-ray Source	2711 reflections with *I* > 2σ(*I*)
Graphite monochromator	*R*_int_ = 0.024
Detector resolution: 16.1827 pixels mm^-1^	θ_max_ = 25.0°, θ_min_ = 3.1°
ω scans	*h* = −10→10
Absorption correction: multi-scan (CrysAlisPro; Rigaku OD, 2024)	*k* = −13→13
*T*_min_ = 0.806, *T*_max_ = 1.000	*l* = −16→14
9706 measured reflections	

### (Z)-N-(Prop-2-en-1-yl)-3-(prop-2-en-1-ylimino)isoindol-1-amine Refinement

**Table:**

Refinement on *F*^2^	118 restraints
Least-squares matrix: full	Hydrogen site location: inferred from neighbouring sites
*R*[*F*^2^ > 2σ(*F*^2^)] = 0.059	H-atom parameters constrained
*wR*(*F*^2^) = 0.190	*w* = 1/[σ^2^(*F*_o_^2^) + (0.1125*P*)^2^ + 0.0092*P*] where *P* = (*F*_o_^2^ + 2*F*_c_^2^)/3
*S* = 1.05	(Δ/σ)_max_ < 0.001
4463 reflections	Δρ_max_ = 0.32 e Å^−^^3^
324 parameters	Δρ_min_ = −0.25 e Å^−^^3^

### (Z)-N-(Prop-2-en-1-yl)-3-(prop-2-en-1-ylimino)isoindol-1-amine Special details

**Table:**

Geometry. All esds (except the esd in the dihedral angle between two l.s. planes) are estimated using the full covariance matrix. The cell esds are taken into account individually in the estimation of esds in distances, angles and torsion angles; correlations between esds in cell parameters are only used when they are defined by crystal symmetry. An approximate (isotropic) treatment of cell esds is used for estimating esds involving l.s. planes.
Refinement. Refinement of F^2^ against ALL reflections. The weighted R-factor wR and goodness of fit S are based on F^2^, conventional R-factors R are based on F, with F set to zero for negative F^2^. The threshold expression of F^2^ > 2σ(F^2^) is used only for calculating R-factors(gt) etc. and is not relevant to the choice of reflections for refinement. R-factors based on F^2^ are statistically about twice as large as those based on F, and R- factors based on ALL data will be even larger.

### (Z)-N-(Prop-2-en-1-yl)-3-(prop-2-en-1-ylimino)isoindol-1-amine Fractional atomic coordinates and isotropic or equivalent isotropic displacement parameters (Å^2^)

**Table:**

	*x*	*y*	*z*	*U*_iso_*/*U*_eq_	Occ. (<1)
C1A	0.8690 (3)	0.2668 (3)	0.87377 (17)	0.0533 (6)	
C2A	1.0230 (3)	0.1639 (3)	0.92144 (17)	0.0498 (6)	
C3A	1.1469 (3)	0.0757 (3)	0.8868 (2)	0.0654 (7)	
H3A	1.145361	0.072323	0.820719	0.078*	
C4A	1.2744 (3)	−0.0079 (3)	0.9542 (2)	0.0761 (8)	
H4A	1.360437	−0.067995	0.932452	0.091*	
C5A	1.2771 (3)	−0.0045 (3)	1.0520 (2)	0.0761 (8)	
H5A	1.364199	−0.062658	1.095489	0.091*	
C6A	1.1526 (3)	0.0840 (3)	1.08696 (19)	0.0625 (7)	
H6A	1.154191	0.086940	1.153171	0.075*	
C7A	1.0260 (3)	0.1675 (2)	1.02036 (16)	0.0498 (6)	
C8A	0.8735 (3)	0.2710 (3)	1.02921 (16)	0.0501 (6)	
C9A	0.6704 (11)	0.4090 (15)	0.7545 (8)	0.0947 (15)	0.372 (7)
H9AA	0.673228	0.485475	0.773082	0.114*	0.372 (7)
H9AB	0.592084	0.380871	0.792578	0.114*	0.372 (7)
C9C	0.6654 (5)	0.3983 (8)	0.7465 (6)	0.0947 (15)	0.628 (7)
H9CA	0.626343	0.445630	0.796838	0.114*	0.628 (7)
H9CB	0.597534	0.351483	0.741757	0.114*	0.628 (7)
C10A	0.6249 (14)	0.4511 (9)	0.6435 (8)	0.0758 (15)	0.372 (7)
H10A	0.639887	0.390099	0.606722	0.091*	0.372 (7)
C10C	0.6553 (7)	0.5003 (8)	0.6464 (4)	0.0758 (15)	0.628 (7)
H10C	0.738294	0.527023	0.625014	0.091*	0.628 (7)
C11A	0.561 (2)	0.5848 (9)	0.6032 (16)	0.118 (2)	0.372 (7)
H11A	0.548419	0.641511	0.643313	0.142*	0.372 (7)
H11B	0.528734	0.621055	0.535090	0.142*	0.372 (7)
C11C	0.5214 (10)	0.5504 (9)	0.5897 (8)	0.118 (2)	0.628 (7)
H11C	0.440144	0.521994	0.612675	0.142*	0.628 (7)
H11D	0.509519	0.613615	0.527226	0.142*	0.628 (7)
C12A	0.6832 (3)	0.4077 (3)	1.1195 (2)	0.0706 (8)	
H12A	0.657782	0.388953	1.189409	0.085*	0.755 (5)
H12B	0.604924	0.397485	1.081890	0.085*	0.755 (5)
H12C	0.640908	0.385769	1.184547	0.085*	0.245 (5)
H12D	0.607482	0.423377	1.066716	0.085*	0.245 (5)
C13A	0.6637 (5)	0.5536 (3)	1.0836 (4)	0.0832 (13)	0.755 (5)
H13A	0.563932	0.620544	1.073333	0.100*	0.755 (5)
C13C	0.7305 (18)	0.5270 (9)	1.1046 (15)	0.0832 (13)	0.245 (5)
H13C	0.828096	0.501113	1.136619	0.100*	0.245 (5)
C14A	0.7880 (6)	0.5875 (6)	1.0664 (4)	0.1021 (16)	0.755 (5)
H14A	0.887964	0.520776	1.076508	0.123*	0.755 (5)
H14B	0.774927	0.677829	1.044197	0.123*	0.755 (5)
C14C	0.671 (2)	0.6595 (10)	1.0582 (12)	0.1021 (16)	0.245 (5)
H14C	0.573313	0.698699	1.023136	0.123*	0.245 (5)
H14D	0.726839	0.712478	1.060940	0.123*	0.245 (5)
N1A	0.7836 (2)	0.3261 (2)	0.94508 (13)	0.0565 (6)	
N2A	0.8244 (2)	0.2976 (2)	0.78095 (15)	0.0697 (7)	
N3A	0.8308 (2)	0.3045 (2)	1.11342 (14)	0.0602 (6)	
H3AA	0.893866	0.262979	1.166402	0.072*	
C1B	0.9921 (3)	0.7527 (2)	0.62943 (17)	0.0500 (6)	
C2B	1.1034 (3)	0.8152 (2)	0.58141 (17)	0.0504 (6)	
C3B	1.1871 (3)	0.8759 (3)	0.6156 (2)	0.0641 (7)	
H3B	1.182357	0.881374	0.681332	0.077*	
C4B	1.2780 (3)	0.9283 (3)	0.5487 (2)	0.0741 (8)	
H4B	1.335851	0.969657	0.569866	0.089*	
C5B	1.2853 (3)	0.9209 (3)	0.4514 (2)	0.0777 (9)	
H5B	1.348551	0.956736	0.408388	0.093*	
C6B	1.2003 (3)	0.8612 (3)	0.4158 (2)	0.0658 (7)	
H6B	1.203870	0.857206	0.349727	0.079*	
C7B	1.1099 (3)	0.8079 (2)	0.48381 (17)	0.0517 (6)	
C8B	1.0025 (3)	0.7416 (2)	0.47508 (16)	0.0502 (6)	
C9B	0.8490 (3)	0.6725 (3)	0.75785 (19)	0.0697 (8)	
H9BA	0.860705	0.640276	0.830822	0.084*	
H9BB	0.873532	0.595217	0.732486	0.084*	
C10B	0.6827 (3)	0.7664 (3)	0.7253 (2)	0.0769 (9)	
H10B	0.655008	0.799253	0.656490	0.092*	
C11B	0.5723 (4)	0.8062 (4)	0.7867 (2)	0.1039 (12)	
H11E	0.595419	0.775551	0.855943	0.125*	
H11F	0.471758	0.864697	0.760468	0.125*	
C12B	0.8705 (3)	0.6500 (3)	0.3842 (2)	0.0742 (8)	
H12E	0.880430	0.579368	0.446271	0.089*	
H12F	0.898313	0.608766	0.329684	0.089*	
C13B	0.7045 (3)	0.7495 (3)	0.3651 (2)	0.0847 (9)	
H13B	0.663717	0.791653	0.414733	0.102*	
C14B	0.6106 (5)	0.7831 (5)	0.2852 (2)	0.1256 (16)	
H14E	0.645866	0.743682	0.233435	0.151*	
H14F	0.509075	0.846147	0.280447	0.151*	
N1B	0.9340 (2)	0.7106 (2)	0.55896 (14)	0.0522 (5)	
N2B	0.9588 (2)	0.7386 (2)	0.72120 (14)	0.0602 (6)	
N3B	0.9767 (2)	0.7162 (2)	0.39151 (15)	0.0627 (6)	
H3BA	1.024627	0.739824	0.339276	0.075*	

### (Z)-N-(Prop-2-en-1-yl)-3-(prop-2-en-1-ylimino)isoindol-1-amine Atomic displacement parameters (Å^2^)

**Table:**

	*U*^11^	*U*^22^	*U*^33^	*U*^12^	*U*^13^	*U*^23^
C1A	0.0485 (13)	0.0702 (17)	0.0390 (12)	−0.0187 (12)	0.0054 (9)	−0.0179 (11)
C2A	0.0461 (13)	0.0585 (15)	0.0442 (12)	−0.0187 (11)	0.0069 (9)	−0.0162 (10)
C3A	0.0592 (16)	0.0738 (19)	0.0588 (15)	−0.0158 (14)	0.0124 (12)	−0.0265 (13)
C4A	0.0556 (16)	0.075 (2)	0.0812 (19)	−0.0059 (15)	0.0107 (13)	−0.0251 (15)
C5A	0.0551 (16)	0.082 (2)	0.0680 (17)	−0.0090 (15)	−0.0064 (13)	−0.0085 (15)
C6A	0.0588 (15)	0.0705 (18)	0.0499 (14)	−0.0187 (14)	−0.0028 (11)	−0.0117 (12)
C7A	0.0487 (13)	0.0586 (15)	0.0434 (12)	−0.0224 (12)	0.0049 (9)	−0.0139 (10)
C8A	0.0521 (13)	0.0601 (15)	0.0383 (12)	−0.0211 (12)	0.0053 (10)	−0.0151 (10)
C9A	0.0527 (16)	0.164 (4)	0.0454 (17)	−0.0086 (19)	−0.0001 (12)	−0.0402 (18)
C9C	0.0527 (16)	0.164 (4)	0.0454 (17)	−0.0086 (19)	−0.0001 (12)	−0.0402 (18)
C10A	0.068 (3)	0.076 (4)	0.082 (2)	−0.017 (3)	−0.011 (2)	−0.033 (3)
C10C	0.068 (3)	0.076 (4)	0.082 (2)	−0.017 (3)	−0.011 (2)	−0.033 (3)
C11A	0.133 (6)	0.092 (4)	0.107 (4)	−0.028 (4)	−0.048 (3)	−0.007 (4)
C11C	0.133 (6)	0.092 (4)	0.107 (4)	−0.028 (4)	−0.048 (3)	−0.007 (4)
C12A	0.0700 (18)	0.0767 (18)	0.0576 (16)	−0.0169 (15)	0.0222 (13)	−0.0260 (14)
C13A	0.081 (4)	0.075 (2)	0.074 (3)	−0.013 (2)	0.005 (3)	−0.017 (2)
C13C	0.081 (4)	0.075 (2)	0.074 (3)	−0.013 (2)	0.005 (3)	−0.017 (2)
C14A	0.103 (4)	0.081 (3)	0.111 (3)	−0.034 (3)	0.002 (3)	−0.011 (3)
C14C	0.103 (4)	0.081 (3)	0.111 (3)	−0.034 (3)	0.002 (3)	−0.011 (3)
N1A	0.0505 (11)	0.0710 (14)	0.0400 (11)	−0.0123 (11)	0.0047 (8)	−0.0187 (10)
N2A	0.0534 (12)	0.1011 (18)	0.0439 (12)	−0.0137 (12)	0.0035 (9)	−0.0261 (11)
N3A	0.0615 (13)	0.0763 (15)	0.0399 (11)	−0.0198 (12)	0.0053 (9)	−0.0211 (10)
C1B	0.0462 (13)	0.0520 (14)	0.0451 (12)	−0.0106 (11)	0.0013 (10)	−0.0146 (10)
C2B	0.0408 (13)	0.0518 (15)	0.0506 (13)	−0.0087 (11)	−0.0001 (10)	−0.0145 (11)
C3B	0.0553 (16)	0.0673 (18)	0.0704 (17)	−0.0195 (14)	0.0000 (12)	−0.0251 (14)
C4B	0.0521 (16)	0.0679 (19)	0.102 (2)	−0.0213 (15)	0.0017 (15)	−0.0256 (17)
C5B	0.0523 (16)	0.073 (2)	0.092 (2)	−0.0205 (15)	0.0135 (14)	−0.0051 (16)
C6B	0.0507 (15)	0.0722 (19)	0.0605 (15)	−0.0140 (14)	0.0114 (12)	−0.0120 (13)
C7B	0.0383 (12)	0.0535 (15)	0.0501 (13)	−0.0054 (11)	0.0016 (9)	−0.0113 (11)
C8B	0.0426 (13)	0.0551 (15)	0.0430 (12)	−0.0060 (11)	0.0012 (10)	−0.0161 (11)
C9B	0.0729 (17)	0.085 (2)	0.0475 (14)	−0.0314 (16)	0.0082 (12)	−0.0114 (13)
C10B	0.0752 (18)	0.095 (2)	0.0640 (17)	−0.0407 (18)	0.0056 (14)	−0.0159 (15)
C11B	0.082 (2)	0.143 (4)	0.083 (2)	−0.034 (2)	0.0184 (18)	−0.041 (2)
C12B	0.0806 (19)	0.078 (2)	0.0699 (18)	−0.0240 (16)	0.0003 (14)	−0.0380 (15)
C13B	0.0755 (19)	0.103 (3)	0.085 (2)	−0.0395 (19)	0.0081 (16)	−0.0331 (19)
C14B	0.093 (3)	0.179 (5)	0.097 (3)	−0.047 (3)	−0.007 (2)	−0.030 (3)
N1B	0.0524 (12)	0.0598 (13)	0.0439 (10)	−0.0181 (10)	0.0036 (8)	−0.0182 (9)
N2B	0.0567 (13)	0.0809 (16)	0.0404 (11)	−0.0228 (12)	0.0039 (9)	−0.0183 (10)
N3B	0.0572 (13)	0.0813 (16)	0.0474 (12)	−0.0194 (12)	0.0056 (9)	−0.0252 (11)

### (Z)-N-(Prop-2-en-1-yl)-3-(prop-2-en-1-ylimino)isoindol-1-amine Geometric parameters (Å, º)

**Table:**

C1A—C2A	1.488 (3)	C13C—H13C	0.9300
C1A—N1A	1.396 (3)	C13C—C14C	1.339 (2)
C1A—N2A	1.281 (3)	C14A—H14A	0.9300
C2A—C3A	1.372 (4)	C14A—H14B	0.9300
C2A—C7A	1.387 (3)	C14C—H14C	0.9300
C3A—H3A	0.9300	C14C—H14D	0.9300
C3A—C4A	1.385 (4)	N3A—H3AA	0.8600
C4A—H4A	0.9300	C1B—C2B	1.483 (3)
C4A—C5A	1.372 (4)	C1B—N1B	1.403 (3)
C5A—H5A	0.9300	C1B—N2B	1.275 (3)
C5A—C6A	1.379 (4)	C2B—C3B	1.377 (4)
C6A—H6A	0.9300	C2B—C7B	1.377 (3)
C6A—C7A	1.374 (3)	C3B—H3B	0.9300
C7A—C8A	1.476 (4)	C3B—C4B	1.377 (4)
C8A—N1A	1.317 (3)	C4B—H4B	0.9300
C8A—N3A	1.325 (3)	C4B—C5B	1.373 (4)
C9A—H9AA	0.9700	C5B—H5B	0.9300
C9A—H9AB	0.9700	C5B—C6B	1.386 (4)
C9A—C10A	1.508 (2)	C6B—H6B	0.9300
C9A—N2A	1.473 (2)	C6B—C7B	1.386 (3)
C9C—H9CA	0.9700	C7B—C8B	1.472 (3)
C9C—H9CB	0.9700	C8B—N1B	1.319 (3)
C9C—C10C	1.507 (2)	C8B—N3B	1.322 (3)
C9C—N2A	1.475 (2)	C9B—H9BA	0.9700
C10A—H10A	0.9300	C9B—H9BB	0.9700
C10A—C11A	1.338 (2)	C9B—C10B	1.5012 (19)
C10C—H10C	0.9300	C9B—N2B	1.460 (3)
C10C—C11C	1.337 (2)	C10B—H10B	0.9300
C11A—H11A	0.9300	C10B—C11B	1.3254 (19)
C11A—H11B	0.9300	C11B—H11E	0.9300
C11C—H11C	0.9300	C11B—H11F	0.9300
C11C—H11D	0.9300	C12B—H12E	0.9700
C12A—H12A	0.9700	C12B—H12F	0.9700
C12A—H12B	0.9700	C12B—C13B	1.5006 (19)
C12A—H12C	0.9700	C12B—N3B	1.456 (3)
C12A—H12D	0.9700	C13B—H13B	0.9300
C12A—C13A	1.505 (2)	C13B—C14B	1.3178 (19)
C12A—C13C	1.511 (2)	C14B—H14E	0.9300
C12A—N3A	1.436 (3)	C14B—H14F	0.9300
C13A—H13A	0.9300	N3B—H3BA	0.8600
C13A—C14A	1.330 (2)		
			
N1A—C1A—C2A	109.48 (19)	C13A—C14A—H14A	120.0
N2A—C1A—C2A	124.8 (2)	C13A—C14A—H14B	120.0
N2A—C1A—N1A	125.7 (2)	H14A—C14A—H14B	120.0
C3A—C2A—C1A	133.3 (2)	C13C—C14C—H14C	120.0
C3A—C2A—C7A	120.8 (2)	C13C—C14C—H14D	120.0
C7A—C2A—C1A	105.9 (2)	H14C—C14C—H14D	120.0
C2A—C3A—H3A	121.3	C8A—N1A—C1A	106.5 (2)
C2A—C3A—C4A	117.4 (3)	C1A—N2A—C9A	112.3 (5)
C4A—C3A—H3A	121.3	C1A—N2A—C9C	118.0 (4)
C3A—C4A—H4A	119.1	C8A—N3A—C12A	122.4 (2)
C5A—C4A—C3A	121.7 (3)	C8A—N3A—H3AA	118.8
C5A—C4A—H4A	119.1	C12A—N3A—H3AA	118.8
C4A—C5A—H5A	119.5	N1B—C1B—C2B	109.3 (2)
C4A—C5A—C6A	121.0 (3)	N2B—C1B—C2B	124.1 (2)
C6A—C5A—H5A	119.5	N2B—C1B—N1B	126.5 (2)
C5A—C6A—H6A	121.2	C3B—C2B—C1B	132.6 (2)
C7A—C6A—C5A	117.6 (3)	C7B—C2B—C1B	106.2 (2)
C7A—C6A—H6A	121.2	C7B—C2B—C3B	121.2 (2)
C2A—C7A—C8A	104.9 (2)	C2B—C3B—H3B	121.2
C6A—C7A—C2A	121.5 (2)	C4B—C3B—C2B	117.5 (3)
C6A—C7A—C8A	133.6 (2)	C4B—C3B—H3B	121.2
N1A—C8A—C7A	113.2 (2)	C3B—C4B—H4B	119.2
N1A—C8A—N3A	123.3 (2)	C5B—C4B—C3B	121.6 (3)
N3A—C8A—C7A	123.5 (2)	C5B—C4B—H4B	119.2
H9AA—C9A—H9AB	108.0	C4B—C5B—H5B	119.3
C10A—C9A—H9AA	109.3	C4B—C5B—C6B	121.5 (3)
C10A—C9A—H9AB	109.3	C6B—C5B—H5B	119.3
N2A—C9A—H9AA	109.3	C5B—C6B—H6B	121.7
N2A—C9A—H9AB	109.3	C7B—C6B—C5B	116.7 (3)
N2A—C9A—C10A	111.6 (6)	C7B—C6B—H6B	121.7
H9CA—C9C—H9CB	107.6	C2B—C7B—C6B	121.6 (2)
C10C—C9C—H9CA	108.7	C2B—C7B—C8B	105.2 (2)
C10C—C9C—H9CB	108.7	C6B—C7B—C8B	133.1 (2)
N2A—C9C—H9CA	108.7	N1B—C8B—C7B	113.1 (2)
N2A—C9C—H9CB	108.7	N1B—C8B—N3B	123.4 (2)
N2A—C9C—C10C	114.3 (5)	N3B—C8B—C7B	123.4 (2)
C9A—C10A—H10A	123.3	H9BA—C9B—H9BB	107.9
C11A—C10A—C9A	113.3 (14)	C10B—C9B—H9BA	109.3
C11A—C10A—H10A	123.3	C10B—C9B—H9BB	109.3
C9C—C10C—H10C	121.6	N2B—C9B—H9BA	109.3
C11C—C10C—C9C	116.8 (7)	N2B—C9B—H9BB	109.3
C11C—C10C—H10C	121.6	N2B—C9B—C10B	111.7 (2)
C10A—C11A—H11A	120.0	C9B—C10B—H10B	117.4
C10A—C11A—H11B	120.0	C11B—C10B—C9B	125.2 (3)
H11A—C11A—H11B	120.0	C11B—C10B—H10B	117.4
C10C—C11C—H11C	120.0	C10B—C11B—H11E	120.0
C10C—C11C—H11D	120.0	C10B—C11B—H11F	120.0
H11C—C11C—H11D	120.0	H11E—C11B—H11F	120.0
H12A—C12A—H12B	106.9	H12E—C12B—H12F	108.1
H12C—C12A—H12D	109.4	C13B—C12B—H12E	109.5
C13A—C12A—H12A	107.3	C13B—C12B—H12F	109.5
C13A—C12A—H12B	107.3	N3B—C12B—H12E	109.5
C13C—C12A—H12C	111.6	N3B—C12B—H12F	109.5
C13C—C12A—H12D	111.6	N3B—C12B—C13B	110.7 (2)
N3A—C12A—H12A	107.3	C12B—C13B—H13B	116.7
N3A—C12A—H12B	107.3	C14B—C13B—C12B	126.5 (3)
N3A—C12A—H12C	111.6	C14B—C13B—H13B	116.7
N3A—C12A—H12D	111.6	C13B—C14B—H14E	120.0
N3A—C12A—C13A	120.0 (3)	C13B—C14B—H14F	120.0
N3A—C12A—C13C	100.9 (7)	H14E—C14B—H14F	120.0
C12A—C13A—H13A	119.7	C8B—N1B—C1B	106.1 (2)
C14A—C13A—C12A	120.5 (4)	C1B—N2B—C9B	117.6 (2)
C14A—C13A—H13A	119.7	C8B—N3B—C12B	122.8 (2)
C12A—C13C—H13C	111.2	C8B—N3B—H3BA	118.6
C14C—C13C—C12A	137.6 (13)	C12B—N3B—H3BA	118.6
C14C—C13C—H13C	111.2		
			
C1A—C2A—C3A—C4A	−179.8 (3)	N3A—C8A—N1A—C1A	179.2 (2)
C1A—C2A—C7A—C6A	179.7 (2)	N3A—C12A—C13A—C14A	−14.3 (7)
C1A—C2A—C7A—C8A	0.7 (2)	N3A—C12A—C13C—C14C	142 (2)
C2A—C1A—N1A—C8A	1.6 (3)	C1B—C2B—C3B—C4B	−177.9 (2)
C2A—C1A—N2A—C9A	−176.4 (9)	C1B—C2B—C7B—C6B	178.1 (2)
C2A—C1A—N2A—C9C	177.6 (5)	C1B—C2B—C7B—C8B	0.1 (2)
C2A—C3A—C4A—C5A	0.6 (4)	C2B—C1B—N1B—C8B	0.7 (3)
C2A—C7A—C8A—N1A	0.3 (3)	C2B—C1B—N2B—C9B	−178.8 (2)
C2A—C7A—C8A—N3A	179.9 (2)	C2B—C3B—C4B—C5B	0.2 (4)
C3A—C2A—C7A—C6A	0.2 (4)	C2B—C7B—C8B—N1B	0.4 (3)
C3A—C2A—C7A—C8A	−178.8 (2)	C2B—C7B—C8B—N3B	−180.0 (2)
C3A—C4A—C5A—C6A	−0.6 (5)	C3B—C2B—C7B—C6B	−0.1 (4)
C4A—C5A—C6A—C7A	0.4 (4)	C3B—C2B—C7B—C8B	−178.0 (2)
C5A—C6A—C7A—C2A	−0.2 (4)	C3B—C4B—C5B—C6B	0.5 (4)
C5A—C6A—C7A—C8A	178.6 (3)	C4B—C5B—C6B—C7B	−0.9 (4)
C6A—C7A—C8A—N1A	−178.6 (2)	C5B—C6B—C7B—C2B	0.7 (4)
C6A—C7A—C8A—N3A	1.1 (4)	C5B—C6B—C7B—C8B	178.0 (3)
C7A—C2A—C3A—C4A	−0.5 (4)	C6B—C7B—C8B—N1B	−177.3 (3)
C7A—C8A—N1A—C1A	−1.2 (3)	C6B—C7B—C8B—N3B	2.4 (4)
C7A—C8A—N3A—C12A	178.5 (2)	C7B—C2B—C3B—C4B	−0.4 (4)
C10A—C9A—N2A—C1A	175.7 (11)	C7B—C8B—N1B—C1B	−0.7 (3)
C10C—C9C—N2A—C1A	137.2 (6)	C7B—C8B—N3B—C12B	179.8 (2)
C13A—C12A—N3A—C8A	−81.7 (4)	C10B—C9B—N2B—C1B	−78.5 (3)
C13C—C12A—N3A—C8A	−96.9 (8)	C13B—C12B—N3B—C8B	82.1 (3)
N1A—C1A—C2A—C3A	178.0 (3)	N1B—C1B—C2B—C3B	177.3 (3)
N1A—C1A—C2A—C7A	−1.4 (3)	N1B—C1B—C2B—C7B	−0.5 (3)
N1A—C1A—N2A—C9A	1.8 (9)	N1B—C1B—N2B—C9B	−0.1 (4)
N1A—C1A—N2A—C9C	−4.2 (6)	N1B—C8B—N3B—C12B	−0.6 (4)
N1A—C8A—N3A—C12A	−1.9 (4)	N2B—C1B—C2B—C3B	−3.8 (4)
N2A—C1A—C2A—C3A	−3.6 (5)	N2B—C1B—C2B—C7B	178.4 (2)
N2A—C1A—C2A—C7A	177.0 (2)	N2B—C1B—N1B—C8B	−178.2 (2)
N2A—C1A—N1A—C8A	−176.8 (2)	N2B—C9B—C10B—C11B	−116.7 (4)
N2A—C9A—C10A—C11A	−137.5 (15)	N3B—C8B—N1B—C1B	179.7 (2)
N2A—C9C—C10C—C11C	149.7 (9)	N3B—C12B—C13B—C14B	117.5 (4)

### (Z)-N-(Prop-2-en-1-yl)-3-(prop-2-en-1-ylimino)isoindol-1-amine Hydrogen-bond geometry (Å, º)

**Table:**

*D*—H···*A*	*D*—H	H···*A*	*D*···*A*	*D*—H···*A*
C10*A*—H10*A*···C6*B*^i^	0.93	2.74	3.571 (12)	149
N3*A*—H3*AA*···N2*B*^ii^	0.86	2.11	2.895 (3)	151
N3*B*—H3*BA*···N2*A*^i^	0.86	2.15	2.951 (3)	154

Symmetry codes: (i) −*x*+2, −*y*+1, −*z*+1; (ii) −*x*+2, −*y*+1, −*z*+2.
